# Screening Depression and Related Conditions via Text Messaging Versus Interview Assessment: Protocol for a Randomized Study

**DOI:** 10.2196/12392

**Published:** 2019-03-29

**Authors:** Haomiao Jin, Shinyi Wu

**Affiliations:** 1 Department of Adult Mental Health and Wellness Suzanne Dworak-Peck School of Social Work University of Southern California Los Angeles, CA United States; 2 Edward R Roybal Institute on Aging University of Southern California Los Angeles, CA United States; 3 Daniel J Epstein Department of Industrial and Systems Engineering Viterbi School of Engineering University of Southern California Los Angeles, CA United States

**Keywords:** depression, diabetes, comorbidity, screening, primary care, health information technology, information and communication technology, text messaging, patient-reported outcome measures

## Abstract

**Background:**

Depression is an often underdiagnosed and, therefore, untreated comorbidity for low-income, racially or ethnically diverse patients with a chronic illness such as diabetes. Recent updates from the US Preventive Services Task Force guidelines in 2016 recommend depression screening for every adult but does not suggest the mode of assessment. Short message service (SMS) text messaging is an inexpensive, private, and scalable approach to provide depression screening and monitoring; it can also alleviate many barriers, such as transportation, childcare, and clinical visit time faced by the low-income population, in receiving a diagnosis of depression. Current evidence is inconsistent in comparing technology-mediated assessment versus interviewer (INTW) assessment in collecting sensitive health information, as some studies suggest that technology encourages self-disclosure while the other studies show the opposite effect.

**Objective:**

The proposed study will test the use of SMS text messaging to assess depression and its related conditions, including functional disability, pain, and anxiety, in low-income, culturally diverse, safety-net primary care populations with diabetes. The study will examine the concordance between SMS text message and interviewer assessments and evaluate test-retest reliability.

**Methods:**

The proposed study will adopt a randomized design with 200 patients assigned to four study groups: SMS/INTW, INTW/SMS, SMS/SMS, and INTW/INTW. The first two groups will be used to examine the concordance between SMS text message and interviewer assessments. The third and fourth groups will be used to evaluate test-retest reliability. Participants of the study will be recruited from the participants of the prior Diabetes-Depression Care-management Adoption Trial, a large comparative effectiveness research trial in collaboration with the Los Angeles County Department of Health Services. Test-retest reliability and concordance between SMS text message and interviewer assessments will be evaluated by the interclass correlation coefficient and the kappa statistic. Missing data patterns will be explored to understand whether participants are willing to self-disclose information related to depression in SMS text message assessments.

**Results:**

Recruitment of participants was conducted from June 2017 to November 2017. A total of 206 participants were enrolled: 52 (25.2%) in SMS/INTW, 53 (25.7%) in SMS/SMS, 49 (23.8%) in INTW/SMS, and 52 (25.2%) in INTW/INTW. The average age of the participants was 57.1 years (SD 9.2). A total of 57.8% (119/206) of participants were female, 93.2% (192/206) were Latino, and 77.7% (160/206) chose Spanish as their preferred language. Analysis of the SMS text message assessment shows the cost of distributing the 16 questions is about US $0.50 per person per assessment. Full results of the study will be reported elsewhere.

**Conclusions:**

This study is anticipated to establish the feasibility of using SMS text messaging to assess depression and its related conditions in low-income, culturally diverse, safety-net primary care populations with diabetes. We also expect to generate knowledge about whether patients in the targeted population are willing to reply and self-disclose sensitive information about depression and its related conditions through SMS text message assessments.

**International Registered Report Identifier (IRRID):**

DERR1-10.2196/12392

## Introduction

Depression is an underdiagnosed comorbidity for those with chronic illness [1] that impairs functional status and worsens outcomes, including morbidity, mortality, and health care costs [2-5]. Diabetes doubles the risk of depression relative to the general population [6,7], but as high as 50% of comorbid depression in patients with diabetes is undiagnosed and thus untreated [1]. Low-income, ethnically diverse patients with chronic illnesses are exposed to even higher risk of depression [7-9]. Hispanics have a higher prevalence of diabetes compared with non-Hispanic whites [10] and are less likely to meet hemoglobin A1c and cholesterol goals [11]. Hispanics are less than half as likely as non-Hispanic whites to receive any depression care, including guideline-level depression care [12].

Depression screening is an effective approach to reduce the rate of undiagnosed depression and thus provides timely treatment for patients [13]. As growing evidence suggests the benefits from depression screening are significant and the harms are minimal, the US Preventive Services Task Force updated its guidelines in 2016 that, for the first time in history, recommend providing screening of depression for every adult [13].

Still, significant barriers exist in adopting depression screening among low-income, ethnically diverse patients with chronic illness. This patient population often prefers safety-net primary care over specialty psychiatric care to seek mental health care. However, safety-net primary care providers often find themselves lacking the time and resources to address mental health issues on top of managing other medical conditions like diabetes [14-17]. Concurrently, minority patients are less likely to voluntarily report depressive symptoms, may view depression as a moral weakness or character flaw (ie, not an illness), and may be more likely to ascribe symptoms of depression to a physical illness [18]. Therefore, low-income, predominantly minority patients in safety-net care systems often miss out on screening and diagnosis for depression [15,19].

The proposed study will test the use of short message service (SMS) text messaging to assess depression and its related conditions, including functional disability, pain, and anxiety, in low-income, ethnically and racially diverse safety-net primary care populations. Text messaging is a highly prevalent and global phenomenon; among the 4 billion mobile phones in use, 3.05 billion, or 75%, are SMS enabled [20]. In the United States, texting among adult mobile phone users is higher among Hispanics and Latinos (83%) than among non-Hispanic whites (70%) [21]. SMS text messaging is also inexpensive, private, and can be scaled to large populations [20,21]. Thus, SMS text messaging could be an ideal approach for conducting depression screening and monitoring in underserved, ethnically and racially diverse populations.

The proposed study will test the use of SMS text messaging, in both English and Spanish, to administer the 8-item Patient Health Questionnaire (PHQ-8) assessment for depression. The Patient Health Questionnaire (PHQ) is a well-validated and widely used depression assessment tool in primary care and the general population [22-24]. Prior studies have shown that the PHQ can be reliably carried out over the telephone by interviewers [25]. However, to the best or our knowledge, no prior studies have evaluated the assessment of the PHQ using SMS text messaging in a safety-net primary care population. In addition, this is the first study to test SMS text messaging assessment for highly comorbid conditions of depression, including anxiety [26], functional disability [8], and pain [27].

## Methods

### Overall Design

The proposed study aims to recruit 200 patients randomly assigned to one of the four study groups: SMS/interviewer (INTW), INTW/SMS, SMS/SMS, or INTW/INTW. The first two groups will be used to examine the concordance between SMS and interviewer assessments for depression and its related comorbid conditions, including anxiety, functional disability, and pain. The third and fourth groups will be used to evaluate test-retest reliability. Depression assessment will be carried out using the PHQ-8, a widely used depression assessment tool in primary care and general populations [28]. The first two questions of the PHQ-8 are often used for brief assessment of depression and are known as the 2-item PHQ (PHQ-2) [28]. Anxiety will be assessed using the 2-item Generalized Anxiety Disorder scale (GAD-2) [29]. Functional disability will be assessed using the 3-item Sheehan Disability Scale (SDS) [30]. Pain will be assessed by the presence of chronic pain, pain level, and pain interference. Validity of interviewer assessments for the PHQ, SDS, and GAD-2 have been established in prior studies [25,28-30]. By completing the specific aim of this study, we expect to generate data and results that could be used to examine the validity and reliability of SMS text messaging assessment for depression and its related conditions in a safety-net primary care population with diabetes.

### Recruitment

Participants of this study will be recruited from the group of participants from the prior Diabetes-Depression Care-management Adoption Trial (DCAT), a large, US Department of Health and Human Services-funded translational study conducted from 2010 to 2013 [15,31-37]. These patients will be chosen from the DCAT because of the prior contacts and rapport built during the DCAT so that this study can fit within the short timeline of the funding requirements. The DCAT was a comparative effectiveness research trial with three study arms in collaboration with eight safety-net primary care clinics of the Los Angeles County Department of Health Services, the second-largest safety-net system in the United States. The DCAT tested an automatic telephone depression-monitoring system to facilitate collaborative depression care in patients with type 2 diabetes. A bilingual recruiter and interviewer from the DCAT will join the proposed study and will contact the participants in the DCAT technology-facilitated care (TC) group for recruitment. Participants in the DCAT TC group received regular depression monitoring every 3 or 6 months over automatic telephone calls during the DCAT. For those who agree to participate, a random number generator will be used to randomize the patients into one of the four study groups. Participants assigned to the INTW/SMS and INTW/INTW groups will receive assessments over the telephone immediately after they agree to participate. Participants assigned to the SMS/INTW and SMS/SMS groups will be notified that an SMS text message assessment will be sent to them within 48 hours.

### Sample Size Determination

Unfortunately, there is no consensus in methods to determine the sample size a priori for studying concordance, validity, and reliability [38,39]. Well-received published studies that evaluated the PHQ and SDS in primary care with interviewer assessment typically had a sample size that ranged from 100 to over 3000 [22-23,25,30]. A widely cited study that evaluated the concordance and test-retest reliability of interviewer assessments carried out over the telephone versus in person for the PHQ had a sample size about 350 [25]. Based on the sample size of previous studies, we suggest that a sample size of 200 would be appropriate, given the limit of our funding, while we acknowledge that a sample size over 1000 would be ideal to produce more reliable results. This study will aim to recruit and study 50 patients in each of the four study groups. As mentioned above, we will first recruit patients from the DCAT TC group (N=442), since these patients are more familiar with technology-mediated assessment for the PHQ. If the number of participants recruited from the TC group is smaller than the targeted sample size (ie, 50 in each of the four study groups), patients will be recruited from the other two study arms in the DCAT, namely the usual care group (N=484) and the supported care group (N=480).

### Design of the Text Messaging Assessment

**Figure 1:**
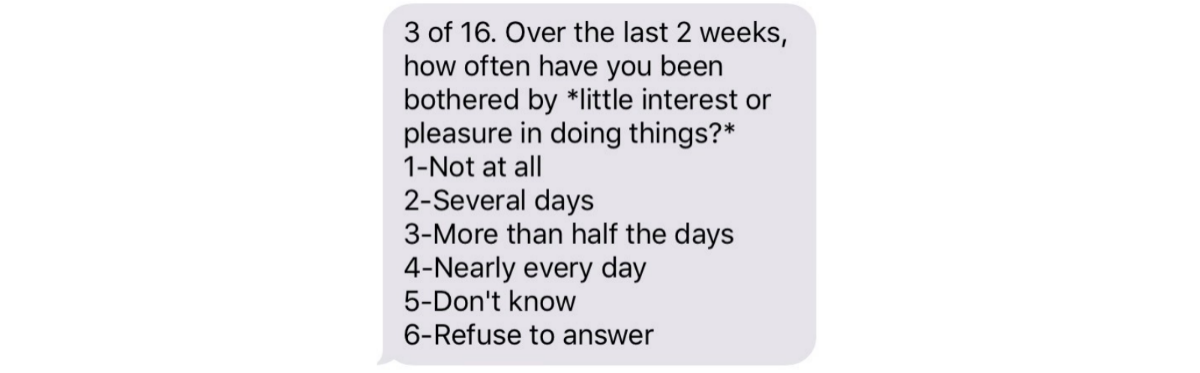
A sample text message for assessing depression and its related conditions. The asterisks highlight the important part of the message.

The SMS text messaging assessment will include 16 questions, delivered in the order of the GAD-2, the PHQ-8, the SDS, and pain assessments (see Table 1). The 16 questions will be sent one at a time. After the participant answers a question by texting back the one number that best describes their feelings, the next message will be sent. The questions are numbered as “1 of 16, 2 of 16...” for participants to track their progress. Since SMS text messaging only supports the delivery of plain text, we use the asterisk sign (ie, *) to highlight the important part of a text message. A sample SMS text messaging assessment question is shown in Figure 1. The SMS text messaging assessment is available in both English and Spanish and implemented using the Qualtrics SMS Distribution module [40].

### Randomization and Data Collection Plan

**Figure 2:**
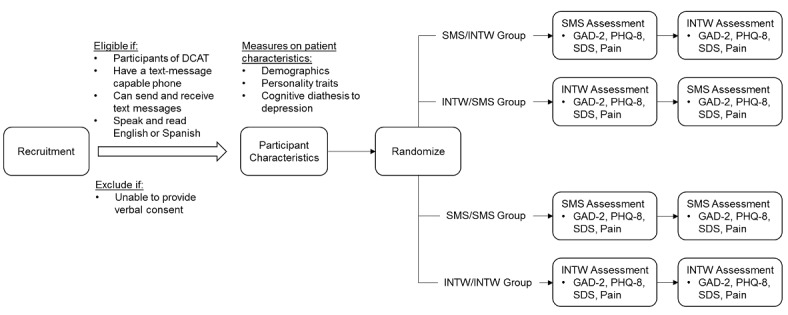
Randomization and data collection plan. DCAT: Diabetes-Depression Care-management Adoption Trial; GAD-2: 2-item Generalized Anxiety Disorder scale; INTW: interviewer; PHQ-8: 8-item Patient Health Questionnaire; SDS: 3-item Sheehan Disability Scale; SMS: short message service.

The randomization and data collection plan is diagramed in Figure 2. After screening for eligibility and obtaining verbal consent to participate, participants will be randomized to one of the four study groups: SMS/INTW, INTW/SMS, SMS/SMS, or INTW/INTW. For all participants, measures on the following characteristics will be collected by a bilingual (ie, English and Spanish) interviewer:

1. Demographics: age, gender, race and ethnicity, language, marital status, education, and insurance.
2. Personality: Ten Item Personality Measure (TIPI) of the Big Five Personality [41].
3. Cognitive diathesis to depression: 9-item Dysfunctional Attitudes Scale (DAS) Short Form [42].
4. Depression stigma: Depression Stigma Scale (DSS) [43].
5. Mobile phone, Internet, and social media use.
6. Depression history and treatment.

The above participant characteristics will be measured because evidence has suggested that demographics such as gender, personality dimensions such as extraversion, dysfunctional attitudes that exist in individuals with depression, and technology use can influence self-disclosure of sensitive health information [44-46]. Measuring these characteristics is critical for determining the generalizability of the proposed study.

Following the collection of characteristics, participants in the SMS/INTW group will first receive the SMS text message assessment within 48 hours. Within 7 days following the SMS text message assessment, the interviewer will contact the participant over the telephone to repeat the same assessment. Participants in the INTW/SMS group will first answer the interviewer assessment over the telephone; they will then reply to the SMS text message assessment within a 7-day period. Participants in the SMS/SMS and INTW/INTW groups will receive two SMS text messages and two interviewer assessments, respectively, within a 7-day period. The choice of interval between the two assessments (ie, 7 days) is based on a prior study that compares interviewer assessment carried out over the telephone versus in-person assessment of depression [25]. A shorter interval is likely to increase the likelihood of repeating the answer from the first assessment in the subsequent one, while a longer interval will increase the probability of change in real severity of depression.

**Table 1:** Text messages and their response options for assessing depression and related conditions. Asterisks highlight the important part of the text message.

Module and text message		Response options
**2-item Generalized Anxiety Disorder scale**		
	1 of 16. Over the last 2 weeks, how often have you been bothered by *feeling nervous, anxious, or on edge?*	1: Not at all; 2: Several days; 3: More than half the days; 4: Nearly every day; 5: Don’t know; 6: Refuse to answer
	2 of 16. Over the last 2 weeks, how often have you been bothered by *not being able to stop or control worrying?*	1: Not at all; 2: Several days; 3: More than half the days; 4: Nearly every day; 5: Don’t know; 6: Refuse to answer
**8-item Patient Health Questionnaire**		
	3 of 16. Over the last 2 weeks, how often have you been bothered by *little interest or pleasure in doing things?*	1: Not at all; 2: Several days; 3: More than half the days; 4: Nearly every day; 5: Don’t know; 6: Refuse to answer
	4 of 16. Over the last 2 weeks, how often have you been bothered by *feeling down, depressed, or hopeless?*	1: Not at all; 2: Several days; 3: More than half the days; 4: Nearly every day; 5: Don’t know; 6: Refuse to answer
	5 of 16. Over the last 2 weeks, how often have you been bothered by *trouble falling or staying asleep, or sleeping too much?*	1: Not at all; 2: Several days; 3: More than half the days; 4: Nearly every day; 5: Don’t know; 6: Refuse to answer
	6 of 16. Over the last 2 weeks, how often have you been bothered by *feeling tired or having little energy?*	1: Not at all; 2: Several days; 3: More than half the days; 4: Nearly every day; 5: Don’t know; 6: Refuse to answer
	7 of 16. Over the last 2 weeks, how often have you been bothered by *poor appetite or overeating?*	1: Not at all; 2: Several days; 3: More than half the days; 4: Nearly every day; 5: Don’t know; 6: Refuse to answer
	8 of 16. Over the last 2 weeks, how often have you been bothered by *feeling bad about yourself, or that you are a failure, or have let yourself or your family down?*	1: Not at all; 2: Several days; 3: More than half the days; 4: Nearly every day; 5: Don’t know; 6: Refuse to answer
	9 of 16. Over the last 2 weeks, how often have you been bothered by *trouble concentrating on things, such as reading the newspaper or watching television?*	1: Not at all; 2: Several days; 3: More than half the days; 4: Nearly every day; 5: Don’t know; 6: Refuse to answer
	10 of 16. Over the last 2 weeks, how often have you been bothered by *moving or speaking so slowly that other people could have noticed, or the opposite—being so restless that you have been moving around a lot more than usual?*	1: Not at all; 2: Several days; 3: More than half the days; 4: Nearly every day; 5: Don’t know; 6: Refuse to answer
**3-item Sheehan Disability Scale**		
	11 of 16. On a scale from 0-10, to what extent has your health interfered with your *work*, including school work and housework, in the past month (0: Not at all; 10: Extremely)?	Score from 0-10, where 0 means *Not at all* and 10 means *Extremely*
	12 of 16. On a scale from 0-10, to what extent has your health interfered with your *family life* in the past month (0: Not at all; 10: Extremely)?	Score from 0-10, where 0 means *Not at all* and 10 means *Extremely*
	13 of 16. On a scale from 0-10, to what extent has your health interfered with your *social life* or relationships with others outside your family in the past month (0: Not at all; 10: Extremely)?	Score from 0-10, where 0 means *Not at all* and 10 means *Extremely*
**Pain**		
	14 of 16. Have you had pain that has been present most of the time for 6 months or more during the past year?	1: Yes; 2: No; 3: Don’t know
	15 of 16. During the past 4 weeks, how much did pain interfere with your normal work, including both work outside the home and housework?	1: Not at all; 2: A little bit; 3: Moderately; 4: Quite a bit; 5: Extremely; 6: Don’t know
	16 of 16. Please rate your pain by telling us the one number that best describes your pain at its WORST in the last 24 hours (0: No pain; 10: Pain as bad as you can imagine).	Score from 0-10, where 0 means *No pain* and 10 means *Pain as bad as you can imagine*

### Patient Safety Plan

If patients report a PHQ-8 score of 8 or higher in any one of the assessments, at the end of the assessment week, a text message will be sent to suggest that they speak to their clinician. Although the chance is small, participants may express thoughts about being better off dead, hurting him/herself, or other suicidal ideation at some point during the telephone recruitment or interview. If this occurs, the recruiter or interviewer will say, “I am not a clinician and I am not qualified to evaluate these thoughts and feelings in detail, but it is important that you get proper medical attention and I would encourage you to discuss these thoughts and feelings with a medical or mental health professional.”

### Statistical Analysis

Concordance between SMS text message and interview assessments will be measured by the intraclass correlation coefficient (ICC) and the kappa statistic. ICC measures the consistency or reproducibility of SMS text message and INTW assessments. We will calculate both the consistency and absolute agreement measures of ICC. The kappa statistic measures interrater agreement for categorical items. We will use different threshold levels to compute kappa, including PHQ-2 ≥3 and PHQ-8 ≥8, two threshold levels that were suggested to classify major depression [22,28]. We will also compute paired *t* tests to investigate differences in mean scores between SMS text message and interviewer assessments. ICC and kappa will be computed using the “icc” and “kappa2” functions in the R package “irr” (The R Foundation). Paired *t* tests will be performed by the R function “t.test.” Test-retest reliability of the SMS text message and interviewer assessments will also be measured by ICC. Finally, internal consistency will be measured by Cronbach alpha, which will be computed using the “alpha” function in the R package “psych.”

Since information such as depression and functional disability is sensitive health information, it is critical to understand whether participants are willing to self-disclose such information in an SMS text message assessment. We will calculate the proportion of missing data and explore whether the data are missing at random or is likely to follow some patterns, using descriptive and visualization tools provided by the R packages “mice” and “VIM.” We will also perform multiple imputation on the data and compare the concordance and reliability measures on original and imputed data [47].

## Results

This study was funded by the Research Council of the Suzanne Dworak-Peck School of Social Work at the University of Southern California and was approved by the University of Southern California Institutional Review Board. Recruitment of participants was conducted from June 2017 to November 2017. A total of 769 call attempts were made. Among those attempts, 490 (63.7%) were unsuccessful for the following reasons: phone number disconnected (196/490, 40.0%); left voicemail, but participant did not return call (176/490, 35.9%); wrong number (73/490, 14.9%); no one picked up the phone (28/490, 5.7%); or other reasons (53/490, 10.8%). Of the 769 call attempts, 279 (36.3%) patients were successfully contacted and assessed for eligibility. Of the 279 patients that were contacted, 73 (26.2%) were excluded for the following reasons: does not have or know how to use cell phone and/or text message (44/73, 60%) or declined to participate (29/73, 40%). The recruitment led to the enrollment of 206 participants: 52 (25.2%) in SMS/INTW, 53 (25.7%) in SMS/SMS, 49 (23.8%) in INTW/SMS, and 52 (25.2%) in INTW/INTW. The average age of the participants was 57.1 years (SD 9.2). A total of 57.8% (119/206) of the participants were female, 93.2% (192/206) were Latino, and 77.7% (160/206) chose Spanish as their preferred language. Analysis of the SMS text message assessment shows the cost of distributing the 16 questions was about US $0.50 per person per assessment, which includes all 16 questions. Full results of the study will be reported elsewhere.

## Discussion

This paper proposed a study with a randomized design to analyze SMS text message assessment, in both English and Spanish, for depression and its related conditions in a safety-net primary care population. The US Preventive Services Task Force recommends depression screening for all adults but does not mention the mode of screening [13]. Existing research has tested the PHQ for depression screening using paper-based self-reported assessment [48-50], in-person interviewer assessment [25,51], and telephone interviewer assessment [15,25]. To the best of our knowledge, there is no published paper about how widely each of those assessment modes are used in practice. The DCAT study was conducted in the Los Angeles County Department of Health Services, which carries out depression screening using the PHQ by in-person interviewer assessment. Compared to other assessment modes, SMS text message assessment is inexpensive, private, and scalable in large populations. Such advantages of SMS text message assessment are particularly beneficial for our targeted population in this study (ie, low-income, racially and ethnically diverse, safety-net patients with diabetes). These patients often face more significant individual and systemic barriers to receive timely depression assessment and treatment. Thus, the proposed SMS text message approach, if successful, could be a beneficial addition to primary care for these patients by providing proactive and timely depression assessment.

The proposed study will generate data and results that fill in the gap of current evidence in technology-mediated assessment for sensitive health information such as depressive symptoms. Current evidence on the effect of technology on self-disclosing such information is inconsistent. Some studies suggest that technology-mediated assessment such as SMS text messaging can create an idealized perception of the interviewer and reduce social desirability bias [52]. As a result, technology-mediated assessment can encourage disclosure of sensitive health information [53,54]. In contrast, there is also evidence suggesting technology-mediated assessment discourages disclosure of sensitive information, since the distance and private space created by technology may discourage patients to seek help [55].

The proposed study has a few limitations. First, the sample size is relatively small due to the limit of our funding. Second, the study participants’ experience built in the prior DCAT study may make those participants more familiar with technology-mediated assessment than average people in the targeted study population. Nevertheless, the 4-year interval between the DCAT study, conducted from 2010 to 2013, and this study, conducted in 2017, is long enough that it is likely to decrease the potential influences of DCAT assessment. Finally, the study participants are predominantly Latino, which may limit the generalizability of the study results to other safety-net primary care populations, especially those with a majority of African American patients.

The anticipated outcome of this study is to establish feasibility of using SMS text messaging to assess depression and its related conditions. Specifically, we aim to gather data and results for concordance and test-retest reliability of SMS text message and interviewer assessments in low-income, racially and ethnically diverse populations with diabetes. We also expect to generate preliminary knowledge about whether patients in the targeted population will be willing to reply and self-disclose sensitive health information about depression and its related conditions through SMS text message assessments.

## Abbreviations

DAS: 9-item Dysfunctional Attitudes Scale
DCAT: Diabetes-Depression Care-management Adoption Trial
DSS: Depression Stigma Scale
GAD-2: 2-item Generalized Anxiety Disorder scale
ICC: intraclass correlation coefficient
INTW: interviewer
PHQ: Patient Health Questionnaire
PHQ-2: 2-item Patient Health Questionnaire
PHQ-8: 8-item Patient Health Questionnaire
SDS: 3-item Sheehan Disability Scale
SMS: short message service
TC: technology-facilitated care
TIPI: Ten Item Personality Measure
